# Scrambling for access: availability, accessibility, acceptability and quality of healthcare for lesbian, gay, bisexual and transgender people in South Africa

**DOI:** 10.1186/s12914-017-0124-4

**Published:** 2017-05-30

**Authors:** Alex Müller

**Affiliations:** Gender, Health and Justice Research Unit, University of Cape Town, Room 1.01.5, Health Sciences Faculty, Anzio Rd, Observatory, Cape Town, 7925 South Africa

**Keywords:** Lesbian, gay, bisexual and transgender, Sexual orientation, Gender identity, Right to health, Access to healthcare, Discrimination, General Comment 14, South Africa

## Abstract

**Background:**

Sexual orientation and gender identity are social determinants of health for people identifying as lesbian, gay, bisexual and transgender (LGBT), and health disparities among sexual and gender minority populations are increasingly well understood. Although the South African constitution guarantees sexual and gender minority people the right to non-discrimination and the right to access to healthcare, homo- and transphobia in society abound. Little is known about LGBT people’s healthcare experiences in South Africa, but anecdotal evidence suggests significant barriers to accessing care. Using the framework of the UN International Covenant on Economic, Social and Cultural Rights General Comment 14, this study analyses the experiences of LGBT health service users using South African public sector healthcare, including access to HIV counselling, testing and treatment.

**Methods:**

A qualitative study comprised of 16 semi-structured interviews and two focus group discussions with LGBT health service users, and 14 individual interviews with representatives of LGBT organisations. Data were thematically analysed within the framework of the UN International Covenant on Economic, Social and Cultural Rights General Comment 14, focusing on availability, accessibility, acceptability and quality of care.

**Results:**

All interviewees reported experiences of discrimination by healthcare providers based on their sexual orientation and/or gender identity. Participants recounted violations of all four elements of the UN General Comment 14: 1) Availability: Lack of public health facilities and services, both for general and LGBT-specific concerns; 2) Accessibility: Healthcare providers' refusal to provide care to LGBT patients; 3) Acceptability: Articulation of moral judgment and disapproval of LGBT patients’ identity, and forced subjection of patients to religious practices; 4) Quality: Lack of knowledge about LGBT identities and health needs, leading to poor-quality care. Participants had delayed or avoided seeking healthcare in the past, and none had sought out accountability or complaint mechanisms within the health system.

**Conclusion:**

Sexual orientation and gender identity are important categories of analysis for health equity, and lead to disparities in all four dimensions of healthcare access as defined by General Comment 14. Discriminatory and prejudicial attitudes by healthcare providers, combined with a lack of competency and knowledge are key reasons for these disparities in South Africa.

## Background

Identifying as lesbian, gay, bisexual or transgender (LGBT)^1^ is not genetically or biologically hazardous, but societal homo- and transphobia confer risk factors for the well-being of people who identify as LGBT [1, 2]. While L, G, B, and T are usually tied together as an acronym that suggests homogeneity, each letter represents a wide range of people of different races, classes, ages, socioeconomic status and identities. What unites them as sexual and gender minorities are common experiences of stigma and discrimination, and, specifically with respect to healthcare, a history of pathologisation and discrimination: homosexuality was classified as a mental illness until 1973, and the World Health Organization’s International Classification of Diseases (ICD) retained it for another 20 years until it was removed during an ICD revision in 1992. Gender identity retains a diagnosis in both the American Psychiatric Association’s diagnostic manual DSM-V as well as in WHO’s ICD-10 [3].

As a result, people who identify as LGBT face a common set of challenges in accessing competent health services, and achieving the highest possible level of health [4]. Homophobia, the irrational fear and hatred of people who are attracted to the same sex, and transphobia, the irrational fear and hatred of people who do not fit binary gender identities, lead to social exclusion, experiences of discrimination and stigma, and in the worst case violence directed against people whose real or perceived sexual orientation and gender identity do not fit the narrowly defined heterosexual norms [1, 2, 5, 6]. Gender identity and sexual orientation, like other social determinants of health, lead to health disparities and, compared with heterosexual and cisgender socioeconomically matched peers, individuals who identify as LGBT are more likely to face barriers accessing appropriate healthcare [1, 2]. Beyond these societal risk factors, people who identify as LGBT have specific health and healthcare needs in various fields from chronic disease risk, adult and adolescent mental health, violence, sexually transmitted infections, and human immunodeficiency virus infection [7]. Of special concern are mental health disparities: experiences of social exclusion, discrimination and prejudice impact on mental health, and studies from Europe and the United States have shown that people who identify as LGBT have significantly higher rates of depression, suicide, and anxiety disorders than their heterosexual matched peers [8–10].

These health needs are as urgent in South Africa. While current data focuses on infectious diseases, it confirms findings from other contexts: South African people who identify as LGBT are at higher risk for HIV transmission compared to the general population. Studies with men who have sex with men (MSM) demonstrate high HIV prevalence among these marginalised groups [11, 12]. Similarly, and in contradiction to dominant assumptions about their ‘immunity’, a recent study shows that in Southern Africa, the HIV prevalence among women who have sex with women might be as high as 10% [13]. Almost half of the women in this study reported that they had had heterosexual sex at least once in their life. Even more importantly, one third of these women had experienced sexual violence. These findings complicate the dominant image of women who have sex with women as not at risk for HIV, and indicate that sexual violence is a major risk factor for lesbian, bisexual and gender non-conforming women in South Africa and elsewhere. Findings from the United States suggest that non-heterosexual identities place people at higher risk of experiencing sexual assault, often motivated by homo- or transphobia: a recent review revealed a 43% median estimate of lifetime sexual assault for lesbian and bisexual women in the United States [6]. Indeed, in the past few years, cases of so-called ‘corrective’ rape – sexual violence targeted at gender non-conforming women, often in poor and predominantly black areas – have sparked publicity in South Africa and abroad [14]. These cases are significant reminders that the risks of ‘heterosexual sex’, which are deemed insignificant for lesbian, bisexual and queer women, play out in contexts of homophobia, sexism and misogyny where they acutely shape the HIV risk for these women.

Access to healthcare is challenging in South Africa, a country where the vast majority of the population depend on health services in the under-resourced and overburdened public sector [15]. The private sector, catering for less than 28% of the population [16], accounts for 46% of all health expenditure in the country [17]. Only 16% of South Africans are covered by medical aid [16], the rest of private sector users pay out of pocket and rely on public care for hospitalization [17]. In this highly unequal system, sexual and gender minority people face the general challenges of service and supply unavailability, long waiting times, and a lack of specialized personnel and services, but also encounter homo- and transphobic discrimination and prejudice on top of these other barriers. Recent South African studies highlight that patients identifying as LGBT experience discrimination at the hands of nurses, doctors, counsellors and even administrative and security staff at public health facilities: In a study among men who have sex with men in Soweto, Johannesburg, all respondents recounted experiences of being insulted, ridiculed, or singled out for their sexual orientation [18]. Transgender people seeking access to HIV services routinely experienced being called names or being blamed for acquiring HIV on the grounds of their gender identity [19]. These experiences emphasise that health professionals themselves often act as gatekeepers to services on the ground [20].

The accounts of homo- and transphobia in the health system are not surprising, given that the majority of South Africans believe that homosexuality should not be accepted in society [21]. Justified by arguments calling on ‘tradition’ and ‘culture’, claims that homosexuality is ‘un-African’ are widely accepted, even though they have been widely disproven [22]. Against this widespread homophobia in society, the South African constitution [23] guarantees people who identify as LGBT the right to non-discrimination, including the right to accessing healthcare (Section 9 and 27(a) of the constitution). On policy level, recent years have brought a shift to include LGBT-related health concerns into some South African healthcare policy recommendations. For example, following extensive lobbying by transgender organisations and individuals, transgender people were identified as one of the most-at-risk populations in the 2012–2016 National Strategic Plan for HIV, STIs and TB [24]. Furthermore, civil society groups have begun to call for action as well: the first South African National Health Assembly, held in Cape Town in June 2012, called for ‘appropriate non-judgmental care for marginalised vulnerable groups such as […] LGBT persons’ [25]. While these initiatives to include non-normative identities into health policy are to be welcomed, it remains unclear how these new policies are to be implemented and monitored.

At an international level, treaties and provisions for the right to the highest attainable standard of health acknowledge the impact that social and economic discrimination have on access to and quality of health care. Whilst it makes no mention of sexual orientation or gender identity (probably because it was drafted and adopted in 1954 and 1966 respectively), the International Covenant on Economic, Social and Cultural Rights (ICESCR) defines a number of “other statuses” that can lead to discrimination [26]. Newer documents that add operational definitions to the Covenant make specific mention of sexual orientation, if not gender identity. Paragraph 32 of General Comment 20 on non-discrimination in economic, social and cultural rights [27] includes sexual orientation as one such “other status”, and outlines that “states parties should ensure that a person’s sexual orientation is not a barrier to realising Covenant rights”. General Comment 14 on the highest attainable standard of health defines that access to healthcare, in fulfilment of the requirements for the highest attainable standard of health, consists of four main dimensions: availability, accessibility, acceptability and quality of care [28]. Paragraph 12.b of General Comment 14, which operationalizes the right to health, states that non-discrimination is a key dimension of accessibility to health care; and paragraph 18 explicitly lists sexual orientation in a list of grounds of discrimination and condemns “any discrimination in access to health care and underlying determinants of health, as well as to means and entitlements for their procurement, on the grounds of […] *sexual orientation* […] which has the intention or effect of nullifying or impairing the equal enjoyment or exercise of the right to health” (italics added for emphasis).

Drawing on the four dimensions of access to healthcare specified in General Comment 14 (availability, accessibility, acceptability and quality of care), this paper analyses healthcare for sexual and gender minority South Africans, and places a special emphasis on the impact of discrimination on the grounds of sexual orientation or gender identity on all four levels of healthcare access. It is worthwhile to note that at the time of data collection, South Africa had not yet ratified the ICESCR, and was therefore not bound to implement General Comment 14 (after a long civil society campaign, the country ratified the ICESCR in 2015). Nevertheless, the framework provides a useful lens through which to analyse access to healthcare for people who identify as LGBT, and links discrimination based on sexual orientation and gender identity to broader discourses around the right to health.

## Methods

### Study aim and design

The study explored the healthcare experiences of South African health service users who identify as LGBT, using a qualitative methodology with a strategic snowball sample. Since there is little qualitative data on this topic in South Africa, it was conceptualized as an exploratory study without a specific hypothesis. Rather, it aimed to elicit knowledge following a grounded theory approach. Acknowledging that the experiences of marginalised groups are often excluded from knowledge creation [29], findings were first and foremost based on 16 interviews with health service users who identified as LGBT, supplemented by two focus group discussions with 6 (first group) and 8 (second group)LGBT-identifying health service users. The interviews and focus group discussions were complemented by 14 interviews with representatives from organisations providing services and advocacy to individuals who identify as LGBT, in order to gain a more structural perspective on the individual narratives.

### Recruitment and data collection

LGBT health service users were identified through a snowball convenience sample method, by the use of social media, existing networks of LGBT service organisations and support services, as well as through a website created for the study. People who participated were asked to spread the word to others. The study was approved by the Human Research Ethics Committee of the University of Cape Town’s Faculty of Health Sciences (reference 537/2012), and all participants signed informed consent forms.

Interviews and focus group discussions with health service users who identify as LGBT were based on a thematic guideline that elicited information about people’s use and general experiences in public healthcare facilities, specific incidences of discrimination based on sexual orientation or gender identity, factors that might facilitate or hinder access to care, and participants’ recommendations for the improvement of health services to sexual and gender minorities. The thematic guideline for representatives of organisations was similar but representatives were asked to provide their opinion based on their professional expertise, and provide examples of anonymised cases where possible. Recruitment and data collection continued until data saturation was reached.

Interviews and focus groups discussions were conducted in English, recorded and transcribed. Any words in other South African languages were translated during the transcription process. Based on the four categories of healthcare access defined in the UN General Comment 14 (availability, accessibility, acceptability, quality of care), all transcripts were first read for emerging general themes, and then re-read and coded to identify sub-themes within each of the four categories. Data from the individual interviews and focus group discussions were triangulated in the analysis.

### Participant characteristics and study setting

Of the 16 health service users who participate in individual interviews, ten identified as cisgender gay men, four as cisgender lesbian women, one as cisgender queer woman and one as transgender lesbian woman. Seven participants lived in the wider Cape Town area in the Western Cape province, and nine lived in urban or peri-urban areas near Johannesburg, in Gauteng province. The first focus group was conducted in Pretoria with six gay men, and the second focus group in Cape Town with eight lesbian women. The sample broadly represented the demographic racial characteristics of South African public healthcare users – the majority (27) identified as black, two as white and one as ‘coloured’ (a South African term that describes people of mixed race). The 14 representatives of organisations worked at LGBT advocacy and support organisations and at organisations providing legal support and advice in the two provinces. Because of the relative absence of the experiences of transgender health service users (only one participant identified as transgender), representatives of transgender-specific organisations were sought out for participation to ensure that issues related to gender identity were adequately represented in the data.

## Results

All health service user participants had used public sector facilities in the 3 years prior to being interviewed, either as outpatients or as admitted patients for longer hospital stays. Participants’ reasons for seeking public sector care ranged from routine HIV counselling, testing and treatment to care after suicide attempts and experiences of violence. Fifteen out of the 16 individual interviewees had additionally also used private healthcare when they could afford it, usually because they expected better quality treatment and less discrimination.

### Availability of services

*Functioning public health and health-care facilities, goods and services, as well as programmes, have to be available in sufficient quantity within the State party. The precise nature of the facilities, goods and services will vary depending on numerous factors, including the State party’s developmental level. They will include, however, the underlying determinants of health, such as safe and potable drinking water and adequate sanitation facilities, hospitals, clinics and other health-related buildings, trained medical and professional personnel receiving domestically competitive salaries, and essential drugs, as defined by the WHO Action Programme on Essential Drugs.*

#### (UN ICESCR, general Comment 14, article 12.1)

With regards to the availability of services, participants experienced the same challenges as all public facility users: long waiting times, or services only offered at a considerable distance. One of the focus group participants, a black gay man lamented:

“I had to stand too long in the queue. Oh! Oh my word! That is the worst […] I had to wake up at 5 to be helped at 8 or 8:30 or so […] Yes, the queues are too darn [sic] long.”

In addition to the general non-availability, in the experience of study participants, services for specific health concerns related to their sexual orientation or gender identity were absent at public facilities. For example, one of the black lesbian women participating in the Cape Town focus group highlighted that

“There is no queer protection like the safety pack [HIV and STI prevention material consisting of dental dams, gloves, condoms and lubricant]. I would love to see a dispenser at a clinic”.

None of the participants had received health information targeted at people identifying as LGBT at a public health facility. As a coloured gay man from Cape Town remembers:

“When I discovered that I was gay it’s difficult to get information; because even at the clinics you find pamphlets about TB, about HIV and AIDS and even in the pamphlets themselves, because I remember – I had all of them – for me it was the curiosity that if there is anything mentioned about male to male sex? Nothing at all.”

Representatives from organisations confirmed that LGBT-specific health services were generally not available in public sector facilities. Both in the Western Cape and Gauteng, non-governmental organisations provided certain LGBT-specific health services, usually sexual health concerns (testing and treatment for HIV and other sexually transmitted infections), and only in urban and peri-urban areas. Of note is that while participants mentioned three such clinics that provide services for gay men, no similar specialised service for lesbian women or gender non-conforming people are available. Representatives from organisations also emphasised the lack of gender-affirming services for transgender people. Only three of the tertiary public health facilities in the country provide gender-affirming care, both hormonal and surgical. All three are situated within academic facilities and due to very limited resources, the waiting lists for surgical procedures are up to 20 years long.

Health service user participants confirmed that as a result of the unavailability of information and specialised services, they turned towards non-governmental organisations when they sought knowledge about LGBT-specific health risks and concerns:

“And information […], I do go around and get my information from other sources rather than my clinic, because they do not provide any.” (Black gay man, Johannesburg)

### Accessibility of services

*Health facilities, goods and services have to be accessible to everyone without discrimination, within the jurisdiction of the State party. Accessibility has four overlapping dimensions: non-discrimination, physical accessibility, economic accessibility, information accessibility.*

#### (UN ICESCR, general comment 14, article 12.1)

Accessibility to services came up in numerous interviews as one of the main concerns for health service user participants, and participants focused on the dimension of non-discrimination. At times, barriers to accessibility were as direct as the refusal of services to patients who openly identify as LGBT, as illustrated in this memory from a young black gay men:

“[The nurse said] ‘No, no, go somewhere else, this is not the place for you.’”

However, more often the barriers were subtler than an outright refusal of services, and speak to the manifold ways in which discrimination and prejudice define healthcare experiences for people identifying as LGBT. As one of the black gay men in the Gauteng focus group members succinctly summarised:

“Once we get there [to the clinic], we feel judged”

As one respondent, a white queer woman who lives with a physical and psychosocial disability acknowledged, discrimination based on sexual orientation and gender identity needs to be seen in intersection with other discriminations. According to her experience, public health facilities do not provide additional support for these various markers of difference that place some facility users at disadvantage, and therefore significantly impact their ability to access healthcare:

“It is assumed that you don’t have a physical disability and an emotional disability; and then a mental disability and a hearing disability. And if you do it is assumed that you can fend for yourself and find a way around it.”

### Acceptability of services

*All health facilities, goods and services must be respectful of medical ethics and culturally appropriate,* i.e. *respectful of the culture of individuals, minorities, peoples and communities, sensitive to gender and life-cycle requirements, as well as being designed to respect confidentiality and improve the health status of those concerned.*

#### (UN ICESCR, general comment 14, article 12.1)

Violation of the confidentiality of information about participants’ sexual orientation or gender identity was a common theme in the interviews. Patients’ sexual orientation or gender identity were often shared by healthcare providers with colleagues or other patients. For example, one black lesbian woman recounted that:

“Instead of [the nurse] helping you, they are just going to the others and gossiping that ‘Oh look at them they call themselves lesbians; but they actually sleep with men because if they didn’t’ she wouldn’t be […] positive.’”

Many respondents shared experiences of disrespectful treatment based on their sexual orientation or gender identity. Healthcare providers’ disrespect was usually articulated in verbal harassment: *“They say it’s no good, they make [gay men] a laughing stock”* (black gay man from Gauteng) or disapproving non-verbal cues: “*If we are there they look at us as if we’re sick”* (black gay male focus group participant). Many participants emphasized that such judgmental and discriminatory behaviour was not only perpetuated by healthcare providers, but also by administrative and security staff as well as other patients.

Interviewees recounted various instances of unprofessional behaviour by healthcare providers, for example through intense curiosity and focus on the patients’ sexual orientation or gender identity, as illustrated by the following quote from a black lesbian participant:

“They [healthcare providers] look perplexed in a way that they are a little bit shocked. They ask you ‘So how do you do it [sex]?’ yeah things like that, ‘You really don’t have a man?’ […] it’s not in a professional manner that they are doing it”.

Other participants had experienced religious judgment from healthcare providers. In one recollected incident, a nurse resorted to blaming a young black gay man’s sexual orientation for his suicide attempt:

“[The nurse] is like ‘The reason why you [attempted suicide] is because you are having relationships with men and that is not right. So it’s the evil spirit that is making you do all the things that you are doing.’”

### Quality of care

*As well as being culturally acceptable, health facilities, goods and services must also be scientifically and medically appropriate and of good quality. This requires,* inter alia*, skilled medical personnel, scientifically approved and unexpired drugs and hospital equipment, safe and potable water, and adequate sanitation.*

#### (UN ICESCR, general comment 14, article 12.1)

Healthcare providers’ lack of knowledge about non-normative sexual orientations and gender identities, as well as LGBT-specific health needs was of great concern to all interviewees. As a white queer woman stated:

“The challenge that people face is […] having to explain who they are […] and go through very common myths and preconceptions […] Which is difficult because you go there and you want to seek care, and instead […] you are explaining very basic things to them and you are thinking ‘But why weren’t you taught this in med school?’“^2^

Such lack of knowledge, or existing misinformation, results in exclusion from services. One example was provided by a lesbian women from Cape Town, who was turned away by a nurse when seeking voluntary counselling and testing for HIV, based on the fact that the nurse believed that lesbian women are not at risk for HIV infections. All lesbian interviewees echoed this misconception, summarised by one of the focus group participants:

“To them [nurses] it’s only the straight people that can get HIV […] because straight people they are usually the ones that are sleeping with men”

Representatives from organisations pointed out that the South African Department of Health had not issued to treatment guidelines or algorithms for a number of LGBT-specific health concerns, including gender-affirming care. As a result, there is no nationally agreed-upon standard of care. This lack of national guidance severely compromises the care that LGBT people receive with respect to their specific health concerns. In many instances, healthcare providers’ prejudicial attitudes, and, at times, religiously-motivated bias, also impacted their clinical judgment and formed the basis of inappropriate medical decisions. A young black gay man who sought psychological care after attempting suicide remembered how his psychologist disregarded the usual psychological assessment and treatment approach towards suicidal patients:

“[After learning about my sexual orientation] the psychologist read me some scriptures from the bible; and she told me ‘you know what just pray.’”

### Healthcare users’ reactions

A recurrent theme among both health service user participants and representatives from organisations was the delayed health-seeking behaviour, or the avoidance of health facilities by people who identify as LGBT. The fear of experiencing discrimination, homophobia, or secondary victimisation, combined with an acknowledgement that public facilities often could not provide care for LGBT-specific health concerns constituted significant barriers to accessing care. As a result of health rights violations that they had either experienced themselves, or had heard about from friends and peers, many participants expressed their fear of judgement and discrimination when accessing public health facilities, as one black gay man explained:

“People don’t want to go get their ARVs [antiretroviral treatment] because people are afraid that if I get my ARVs and I’m gay, it’s just gonna be like: ‘You are promiscuous, you deserve it’”

Participants often avoided seeking care at public facilities, as a young black gay man from Johannesburg explained:

“Usually when I get a cold or whatever I nurse myself at home; because of thinking ‘Oh no, but you have to deal with homophobic people, and people whose views are close- minded’ – yeah, I’d rather do it myself, I’d rather nurse myself back to health than deal with them.”

In cases where they could not avoid seeking care at a healthcare facility, participants recounted stories of consciously concealing their sexual orientation or gender identity in order not to experience homo- and transphobia, as the following quote from a black gay male focus group participant illustrates:

“You can’t even tell such things cause… Because when they [nurses] ask ‘Do you have a partner?’ then I say ‘Yes’, and you see that they are inquisitive: “How is she, is she doing you okay?’. [And I will play along and say:] ‘I’ll bring her for a taste’. Because sometimes when you say ‘It’s a he’, it’s like another can of worms is opened: ‘So it is a he, so you sleep with boys? You date, you love other boys?’ and I’m like ‘Oh my god’.”

Despite the diverse health rights violations that participants had experienced when accessing the public health system, the majority of participants did not know about the patients’ rights charter, nor were they aware of what the procedures for laying a complaint about a healthcare provider were:

“In public clinics, you get what you get and you get out. That’s how we are used to it, so I haven’t complained about somebody; but I didn’t even know the procedure to doing so. You know, so I haven’t, and if I was given a chance to, I don’t think I would like to put my word across…” (Black gay man from Pretoria)

Of the few that did have more detailed knowledge on complaints procedures, none had actually filed a complaint. While these service users did recognise that the treatment they received was in violation of their right to health, two reasons emerged that stopped them from reporting the discriminatory incidents. A recurrent opinion was that the line manager of the healthcare providers they lodged a complaint about would not understand the reason for their complaint, or, in the worst case, share the prejudicial attitude of the provider in question. In other words, participants did not trust that the complaint system would provide a satisfactory outcome because they perceived the system to be complacent at best, and prejudicial at worst. A second reason was the fear of retaliatory victimisation at the hands of healthcare providers at subsequent clinic visits. This is an important consideration, especially in areas with less health infrastructure, where service users cannot choose not to attend a certain facility, or not to see a certain healthcare provider.

## Discussion

All LGBT health service users that were interviewed had experienced some form of discrimination based on their sexual orientation or gender identity. These findings confirm what has been reported in previous South African studies [18, 19, 30, 31]. Lane and colleagues [30] documented that all gay men in their study who visited clinics in the Soweto area had been called names, been ridiculed, or had experienced other forms of discrimination. Similarly, 60% of transgender respondents in a study by Stevens [19] had made negative experiences in public clinics. Confirming these quantitative findings, the qualitative findings presented here offer important insights into the nature and impact of discriminatory experiences of LGBT service users in South African public health facilities.

Highlighting issues related to sexual orientation and gender identity in the framework of the UN General Comment 14 shows that these identity categories impact healthcare access on each of the four levels of availability, accessibility, acceptability and quality of care. Using the framework of the UN General Comment 14 operationalisation for realising the highest attainable standard of health provides concrete areas for action in order to improve right to health and access to healthcare for people who identify as LGBT. Interpreting the findings of the study in this framework, in addition to drawing on South African national legislation, can also provide a further impetus for addressing the barriers to healthcare access that participants described. Sexual orientation and gender identity should thus be taken into account when operationalizing the right to health, so that General Comment 14’s potential for achieving health equity for disadvantaged groups [32] also includes groups who are disadvantaged based on their sexual orientation or gender identity.

The findings on the availability of health services are in accordance with studies of access to healthcare among the general population of South Africa [17]. The challenges of a general lack of services, long waiting times, and under-resourced facilities reflect the inequalities in South Africa’s health system, which struggles with a quadruple burden of disease and a serious lack of medical personnel [17]. People who identify as LGBT, like all South Africans who rely on public health facilities, are negatively affected by these resource constraints. However, as the findings from this study show, people who identify as LGBT face homo- and transphobic discrimination on top of these general healthcare access challenges. While the sample of this study was fairly racially homogenous and therefore cannot provide a nuanced analysis of the intersecting impact of race on LGBT-identifying people’s healthcare experiences, it is likely that intersecting identities, based on race, culture or geographical location, further impact healthcare access.

The findings suggest that engaging healthcare providers is a crucial step towards reducing barriers to healthcare access for people who identify as LGBT. Research confirms this study’s findings that negative experiences with healthcare providers contribute to the erosion of a sense of safety in the healthcare system, and as a consequence, LGBT people avoid seeking care [33]. Another South African study quantifies the extent of poor health-seeking behaviour: in the province of the Western Cape, 16% of LGBT people either delayed seeking healthcare for fear of homophobic treatment, or did not seek medical help at all [34]. Yet, healthcare providers in South Africa are bound by professional codes of conduct that prohibit discrimination based on the constitutional provisions – including, among others, gender and sexual orientation. The professional code for physicians is defined by the Health Professions Council of South Africa, and nurses and other allied health workers have to follow the Batho Pele principles [35] which are applicable to all public services in South Africa. Additionally, the Health Professions Act of 1974 [36] stipulates that “A practitioner shall at all times […] respect patient confidentiality, privacy, choices and dignity.” (Section 27(b)). Discussions about how these professional and legal codes apply to patients who identify as LGBT, combined with conversations about how healthcare providers’ own morals and values influence their service provision [20] should be mandatory for all providers. In addition, sensitisation trainings, as well as LGBT health-specific professional development courses can help to challenge discriminatory and judgmental attitudes towards people who identify as LGBT, and build knowledge for providing LGBT-competent care [37]. For this, existing international guidelines such as the guidelines by the Association of American Medical Colleges [38] should be adapted to local contexts, and all trainings should also be incorporated into medical and allied health professions curricula.

It is important to note that all informants for this study lived in urban or peri-urban areas. While participants’ experiences suggest that the health rights of people identifying as LGBT are significantly impacted by homo- and transphobia in these urban and peri-urban facilities, this is likely to be exacerbated in more rural areas. Access to healthcare in general is much more challenging in rural areas, as public health facilities are at greater distances, and less resourced than in urban areas [39]. Furthermore, LGBT people in urban areas have better access to support networks in the form of non-governmental organisations or informal communities, both of which are less likely to existent in rural areas [40]. An important area for future research is to investigate the experiences of people who identify as LGBT and live outside of urban centres, and investigate how sexual orientation and gender identity-related barriers intersect with more ‘general’ barriers linked to geographical location and socio-economic status.

The results from this study also suggest that current complaint and accountability systems are inadequate to capture and respond to complaints by health service users identifying as LGBT. Currently, the South African Department of Health provides complaint hotlines for each province (some of which are toll-free numbers), but encourages patients to complain to the relevant clinic or hospital managers first. The vast majority of participants did not know how to lodge complaints, and even those who did were afraid of encountering further prejudice in the complaint system. In order to improve accountability mechanisms, two important strategies stand out. First, people who facilitate complaint structures need to be aware of and sensitive to sexual orientation- and gender identity-related discrimination. The same strategies that can reduce homophobia among healthcare providers [41] can be employed to sensitise administrative personnel in public health facilities, as well as policy makers and government representatives. Furthermore, the existing accountability mechanisms need to be strengthened, not only to accommodate complaints from health service users who identify as LGBT, but in order to provide better accountability to all health service users. With the introduction of the National Health Insurance policy in South Africa, a number of improvements are foreseen to improve accountability in the public health system. The National Department of Health is to establish a central Office of Standards Compliance, which is meant to act as a central overseeing body to address complaints and grievances by health service users [42]. Such new structures need to acknowledge and address sexual orientation- and gender identity-related discrimination and disparities.

This study is subject to a number of important limitations. The emphasis of the project was on eliciting narratives from a range of health service users who identify as lesbian, gay, bisexual or transgender. As such, it does not seek to portray a universal truth about experiences, but rather highlight specific cases that illustrate shortcomings in the current public health system. The study also did not elicit the perspectives of healthcare providers, and thus cannot confirm healthcare providers’ motivations for the behaviour described by health service users. Other research from South Africa suggests that while some providers do bring their own moral judgments into their patient care, others may simply be unaware of specific health concerns related to sexual orientation and gender identity [40]. Second, the interviews for this study were conducted in the Western Cape and Gauteng, the two wealthiest provinces that have the best health infrastructure in South Africa. This was done on purpose, as the infrastructure in these two provinces provides some support network for people who identify as LGBT. Given that the interviews could have resurfaced traumatic events for participants, they were conducted in areas where participants had access to such LGBT-friendly support services. As already discussed, however, this likely introduced some participant bias. Third, recruitment for the study happened to a large extent through community-based organisations, which could have introduced a selection bias. People who are, in one way or another, in touch with an advocacy and support organisation, might be more likely to have some kind of access to services, either supported or facilitated by these organisations. If the narratives and experiences elicited in this study, which document discriminatory and prejudicial barriers in accessing healthcare, are those of people with better access to services, then the study at best underestimates the challenges that LGBT people face when accessing public health services in South Africa.

## Conclusion

The findings from this study highlight that people who identify as LGBT face numerous challenges when accessing public healthcare in South Africa. While some of these challenges can be attributed to the general lack of resources in the South African public health system, persisting homo- and transphobia among healthcare providers and administrative staff lead to systematic discrimination against people of non-normative sexual orientations and/or gender identities. As a result, people who identify as LGBT, and who already face health disparities based on their sexual orientation and gender identity, lack access to competent and affirmative health services. Including sexual orientation and gender identity in analyses framed by international right to health instruments such as General Comment 14 is essential to document and address these sexual orientation and gender identity-related barriers.

## Abbreviations

ICESCR: International covenant on economic, social and cultural rights
LGBT: Lesbian, gay, bisexual, transgender

## Footnotes

1 I acknowledge that sexual orientation and gender identity are fluid, and encompass more than the LGBT acronym. For example, a growing group of people have re-appropriated the word ‘queer’ to identify their sexual orientation and/or gender identity. However, the majority of the current research evidence that I cite only focuses on LGBT identities, and I have therefore chosen to adhere to this terminology, in the understanding that a single word, or acronym, can never represent a whole group of people.

2 Also quoted in another output from the same research project [43]. A third output from this project is noted in my bibliography [44].
